# Acute Respiratory Distress Syndrome – quo vadis: Innovative und individualisierte Therapieansätze

**DOI:** 10.1007/s00063-025-01273-w

**Published:** 2025-04-22

**Authors:** Nina Buchtele, Thomas Staudinger

**Affiliations:** Universitätsklinik für Innere Medizin I, Intensivstation 13i2, Medizinische Universität Wien, Währinger Gürtel 18–20, 1090 Wien, Österreich

**Keywords:** Atemstörungen, Beatmung, Künstliche Intelligenz, Kontrollierte klinische Studie, Leitlinie, Respiration disorders, Ventilation, Artificial intelligence, Controlled clinical trial, Guideline

## Abstract

Das akute Atemnotsyndrom (ARDS) ist ein heterogenes klinisches Syndrom, das sich durch eine variable Pathophysiologie und unterschiedliche therapeutische Ansätze auszeichnet. Die jüngsten Leitlinien betonen die Bedeutung der Bauchlagerung und der venovenösen extrakorporalen Membranoxygenierung (vv-ECMO) für schwerste Fälle, während routinemäßige Recruitmentmanöver und extrakorporale CO_2_-Eliminationsverfahren nicht mehr empfohlen werden. Um die Personalisierung der ARDS-Therapie weiter voranzutreiben, zeigt die Identifikation von ARDS-Phänotypen mittels „latent class analysis“ vielversprechende Ansätze zur personalisierten Therapie. Zudem könnten adaptive Plattformstudien und auf künstlicher Intelligenz (KI) basierende Entscheidungsunterstützungssysteme die ARDS-Behandlung optimieren. Die zukünftige ARDS-Therapie wird zunehmend individualisiert sein und auf einer verbesserten Patientenstratifizierung, neuen Studiendesigns und dem gezielten Einsatz moderner Technologien basieren. Dieser Artikel fasst die aktuellen Entwicklungen in der ARDS-Therapie zusammen, insbesondere im Hinblick auf individuelle Behandlungsstrategien, neue Studiendesigns und den Einsatz von künstlicher Intelligenz.

## Abstract (en)

Acute respiratory distress syndrome (ARDS) is a heterogeneous clinical syndrome characterized by variable pathophysiology and different therapeutic approaches. Recent guidelines emphasize the importance of prone positioning and venovenous extracorporeal membrane oxygenation (VV-ECMO) for the most severe cases, while routine recruitment maneuvers and extracorporeal CO_2_-removal techniques are no longer recommended. To further advance the personalization of ARDS therapy, the identification of ARDS phenotypes using latent class analysis offers promising approaches for individualized treatment. Additionally, adaptive platform trials and artificial intelligence (AI)-driven decision-support systems may optimize future ARDS management. The future of ARDS treatment is becoming increasingly individualized, based on improved patient stratification, innovative study designs, and the targeted use of modern technologies. This article summarizes recent developments in ARDS therapy, particularly regarding personalized treatment strategies, new study designs, and the application of artificial intelligence.

## Hintergrund

Die meisten der auf der Intensivstation behandelten Erkrankungen sind klinische Syndrome. Entsprechend zeichnen sich diese nicht durch spezifische Biopsiebefunde, genetische Mutationen, mikrobiologische Kulturen oder serologische Tests aus, sondern vielmehr durch eine Sammlung von klinischen Zeichen und Symptomen, die zusammen das Bild einer klinisch erkennbaren Entität ergeben – wie das akute Atemnotsyndrom (Acute Respiratory Distress Syndrome, ARDS; [1]). Infolgedessen sind kritische Krankheitssyndrome von Natur aus heterogen. Dementsprechend scheint ein One-size-fits-all-Konzept zur universellen Therapie des ARDS unpassend zu sein.

Die zukünftige ARDS-Therapie wird also vermutlich durch individualisierte Behandlungsmöglichkeiten maßgeblich beeinflusst werden, wofür Erkenntnisse der letzten Jahre schon vielversprechende Konzepte geschaffen haben.

## Leitlinienupdate – relevante Neuerungen

In den letzten beiden Jahren erschienen 2 Leitlinienupdates der European Society of Intensive Medicine (ESICM; [2]) bzw. der American Thoracic Society (ATS; [3]), die versucht haben, die Therapiestrategien auf Basis der aktuellen Evidenz zusammenzufassen. Ferner wurde im Jahr 2024 eine neue, umfassendere Definition des ARDS basierend auf den bekannten „Berlin“-Kriterien vorgeschlagen [4]. Hier wird die Diagnose der Oxygenierungsstörung des ARDS über die bekannten Kriterien hinaus um die Sauerstoffsättigung (SpO_2_) bzw. die Ratio aus SpO_2_ und inspiratorischer Sauerstofffraktion (F_i_O_2_) erweitert, um auch nichtinvasiv beatmeten Patient:innen bzw. ARDS-Patient:innen, die in Einrichtungen mit limitierten Ressourcen behandelt werden, Rechnung zu tragen. Die erwähnten rezenten Leitlinien unterscheiden sich in einigen Punkten wesentlich. So wird in der ESICM-Leitlinie auch die nichtinvasive Beatmung erwähnt sowie der Subgruppe des mit Coronaviruserkrankung 2019 (COVID-19) assoziierten ARDS (CARDS) Rechnung getragen (z. B. durch die Möglichkeit eines „awake proning“). Die Leitlinie der ATS empfiehlt im Gegensatz zur ESICM zumindest in eingeschränkter Form den Einsatz von Steroiden und Muskelrelaxanzien sowie ein höheres positives endexspiratorisches Druckniveau (PEEP) bei schwerem ARDS. Eine (vereinfachte) Gegenüberstellung der Empfehlungen findet sich in Tab. 1, die einem rezenten Übersichtsartikel zum ARDS entnommen ist [5]. Die wesentlichen Neuerungen für die klinische Praxis betreffen die Erwähnung unterschiedlicher Phänotypen und der damit verbundenen Möglichkeit der individualisierten Behandlungsstrategien durch das ESICM-Expertenpanel (s. im Folgenden). Darüber hinaus zeigen beide Leitlinien Einigkeit hinsichtlich der Bauchlagerung (starke Empfehlung für Patient:innen mit einer Ratio aus Sauerstoffpartialdruck (p_a_O_2_) und F_i_O_2_ < 150) sowie der venovenösen extrakorporalen Membranoxygenierung (vv-ECMO; starke Empfehlung bei schwerstem ARDS). Routinemäßige Recruitmentmanöver (beide Leitlinien) sowie extrakorporale Verfahren zur CO_2_-Eliminations (ESICM) werden nicht mehr empfohlen.

**Tab. 1:** Supportive Therapieoptionen des akuten Atemwegssyndrom (*ARDS*) gemäß Leitlinienempfehlungen. (Aus [5])

Therapieoption	Empfehlungen ESICM 2023 [2]	Empfehlungen ATS 2023 [3]
HFNO/NIV	Keine Empfehlung (bezüglich Mortalitätsreduktion)	–
	HFNO statt konventionelle O_2_-Therapie zur Intubationsvermeidung	
	Evtl. CPAP/NIV statt konventionelle O_2_-Therapie zur Intubationsvermeidung bei COVID-19	
Protektive Beatmung	Niedriges Tidalvolumen (4–8 ml/kg PBW)	V_t_ ≤ 6 ml/kg PBW + P_plat_ ≤ 30 cmH_2_O
PEEP	Keine Empfehlung zur höheren vs. niedrigeren PEEP-Strategie (Mortalität)	Höhere PEEP-Strategie bei moderatem und schwerem ARDS
	Keine Empfehlung zur PEEP-Titration nach Atemmechanik (Mortalität)	
„Driving pressure“	–	–
Recruitmentmanöver	Keine Routinerecruitmentmanöver (Mortalität)	Keine Recruitmentmanöver zur PEEP-Titration
		Keine prolongierten Recruitmentmanöver mit PEEP ≥ 35 cmH_2_O für > 60 s
Bauchlagerung	Empfohlen bei p_a_O_2_-F_i_O_2_-Ratio < 150 und PEEP ≥ 5 cmH_2_O für mindestens 16 konsekutive Stunden	Empfohlen bei p_a_O_2_-F_i_O_2_-Ratio < 150
	„Awake proning“ empfohlen für ARDS bei COVID-19 (Intubationsvermeidung)	„Awake proning“ nicht adressiert
Neuromuskuläre Blockade	Keine kontinuierliche Relaxierung als Routinemaßnahme	Empfohlen in der Frühphase des schweren ARDS (≤ 48 h Beatmung)
		Überlege Beendigung nach 48 h bei Besserung
ECLS	vv-ECMO empfohlen bei schwerem ARDS und ECMO-entry-Kriterien der EOLIA-Studie	vv-ECMO empfohlen bei schwerem ARDS mit p_a_O_2_-F_i_O_2_-Ratio < 80 oder pH < 7,25 und p_a_CO_2_ ≥ 60 mm Hg unter Beachtung von Kontraindikation und nach Ausschöpfung konservativer Therapiemaßnahmen (optimierte protektive Beatmung, Bauchlagerung, neuromuskuläre Blockade)
	ECCO_2_R nicht empfohlen	
Kortikosteroide	–	Empfohlen für mildes, moderates und schweres ARDS
		Nicht empfohlen nach > 14 Tagen Beatmungsdauer

*ATS* American Thoracic Society, *CPAP* „continuous positive airway pressure“, *ECCO*_*2*_*R* „extracorporeal CO_2_ removal“, *ECLS* „extracorporeal life support“, *ESICM* European Society of Intensive Medicine, *F*_*i*_*O*_*2*_ inspiratorische Sauerstofffraktion, *HFNO* „high flow nasal oxygen“, *NIV* nichtinvasive Beatmung, *p*_*a*_*O*_*2*_ Sauerstoffpartialdruck, *PBW* „predicted body weight“, *PEEP* positiver endexspiratorischer Druck, *P*_*plat*_ Plateaudruck,* vv-ECMO* venovenöse extrakorporale Membranoxygenierung, *V*_*t*_ Tidalvolumen

## Indikation zur HFNO-Gabe, NIV und IMV

Die Leitlinie der ESICM empfehlen, die Gabe von „high flow nasal oxygen“ (HFNO) zur Intubationsvermeidung einer konventionellen Sauerstofftherapie vorzuziehen. Hinsichtlich Mortalität wird aufgrund mangelnder Evidenz keine Empfehlung zu HFNO-Gabe bzw. zur nichtinvasiven Beatmung (NIV) gegeben. Die COVID-Pandemie hat allerdings den Pathomechanismus des „patient self inflicted lung injury“ (PSILI) als Ursache einer zusätzlichen progredienten Lungenschädigung bei spontan atmenden Patient:innen in ein breites Bewusstsein gerückt. Durch hohe inspiratorische transpulmonale Drücke bei zunehmender Atemanstrengung mit entsprechender Negativierung des Pleuradrucks kann es zum Atelekttrauma, regionaler Überdehnung und Zunahme des Ödems kommen [6]. Bereits in der großen weltweiten epidemiologischen LUNGSAFE-Studie wurde gezeigt, dass die primäre NIV bei moderat-schwerem ARDS (p_a_O_2_-F_i_O_2_-Ratio < 150) mit einer signifikant erhöhten Mortalität assoziiert war [7]. Auch hier könnte PSILI eine Rolle gespielt haben. Die HFNO-Gabe und die nichtinvasive Beatmung kann also bei ARDS versucht werden, allerdings steigt das Risiko eines Versagens mit zunehmendem Schweregrad der Gasaustauschstörung. Die Detektion von PSILI kann anhand einfacher klinischer Kriterien, wie Atemfrequenz, Atemanstrengung und Gasaustauschstörung, abgeschätzt werden. Spezifischere Kriterien könnten in Zukunft eine individuelle Einschätzung des Risikos einer Fortsetzung der nichtinvasiven Atemunterstützung ermöglichen. Okklusionsverfahren am Respirator wie die Bestimmung des Okklusionsdrucks sind unter NIV zwar möglich, dem Patient:innenkomfort jedoch wenig zuträglich. Die Ösophagusdrucksonde erlaubt die direkte Messung des transpulmonalen Drucks und damit des Risikos für PSILI [8]. Die klassischen Abbruchkriterien der nichtinvasiven Beatmung sollten natürlich ebenfalls beachtet werden. Ein Hinauszögern der Intubation bei Vorliegen von Abbruchskriterien ist jedenfalls unbedingt zu vermeiden. In Tab. 2 findet sich eine Auswahl von in der Literatur beschriebene Risikofaktoren für ein Versagen einer nichtinvasiven Atemunterstützung.

**Tab. 2:** Ausgewählte Risikofaktoren für die Entwicklung eines „patient self inflicted lung injury“ (PSILI). (Modifiziert nach [8] und [6])

Parameter	Berechnung/Beschreibung
Forcierte inspiratorische Atemanstrengung, Atemfrequenz ≥ 30/min	–
Zunahme von Ödem und Konsolidierungen in der Bildgebung	Röntgenuntersuchung, Computertomographie oder Lungenultraschall
Abnahme von C_rs_	C_rs_: Tidalvolumen (in ml)/∆P_stat_
NIV: HACOR-Score > 5 nach 1 h NIV	Herzrate, Acidose, Bewusstsein, Oxygenierung, Atemfrequenzskala
HFNO: ROX-Index < 2,85, < 3,47 und < 3,85 nach 2, 6 und 12 h Therapie	ROX-Index: SpO_2_-F_i_O_2_-Ratio durch Atemfrequenz
P_L_ _insp_ > 20 cmH_2_0	Inspiratorischer transpulmonaler Druck (P_plat_ − P_es_ _insp_)
∆P_L_: > 15–20 cmH_2_O	P_L_ _insp_ − P_L_ _exp_
∆P_es_: > 10–12 cmH_2_0	P_es_ _exp_ − P_es_ _insp_
∆P_occ_ _korr_: > 13–15 cmH_2_O	Inspiratorischer Okklusionsdruck: (PEEP − negativer Atemwegsdruck unter Okklusion) × 0,75
∆P_occ_ _korr_ − ∆P_dyn_ × 0,66 ∶ > 16–17 cmH_2_O	∆P_dyn:_ Dynamischer Driving Pressure

*C*_*rs*_ Compliance des respiratorischen Systems,* ∆P* „driving pressure“, *dyn* dynamisch, *F*_*i*_*O*_*2*_ inspiratorische Sauerstofffraktion, *HFNO* „high flow nasal oxygen“, *NIV* nichtinvasive Beatmung, *SpO*_*2*_ Sauerstoffsättigung, *stat* statistisch, *PEEP* positiver endexspiratorischer Druck *P*_*L*_ transpulmonaler Druck, *insp* inspiratorisch, *exp* expiratorisch, *occ* Okkulusionsdruck, *es* ösophagus

## Anwendung und Ausgestaltung individualisierter Therapiestrategien

Angesichts der Komplexität und Heterogenität des ARDS steht die Individualisierung der therapeutischen Maßnahmen im Interesse der Forschung. Zahlreiche Versuche, Unterkategorien („Phänotypen“) zu definieren und Therapiestrategien dementsprechend maßzuschneidern, wurden und werden unternommen – mit mäßigem Erfolg, was harte outcomeorientierte Endpunkte betrifft. Die intensivmedizinische Therapie des Lungenversagen ist im Wesentlichen eine supportive, die meisten Ansätze zur Individualisierung beschränken sich auf das Konzept einer „bridge to recovery“ mit möglichst wenig „Kollateralschäden“ (z. B.: „ventilator induced lung injury“, VILI). Kausale und in die Pathophysiologie eingreifende neue Therapieansätze sind eher noch Seltenheit. Die Tab. 3 zeigt eine Reihe definierter Phänotypen und mögliche darauf abgestimmte therapeutische Konsequenzen.

**Tab. 3:** Phänotypen des akuten Atemwegssyndroms (*ARDS*). (Modifiziert nach [5])

Phänotyp	Beschreibung	Mögliche Therapieansätze
Schweregrad der Hypoxämie	Ausgedrückt durch die p_a_O_2_-F_i_O_2_-Ratio. Unterschiede in der Mortalität	Bei moderat-schwerem ARDS Bauchlagerung, evtl. Muskelrelaxanzien, bei schwerstem ARDS vv-ECMO
Auslösender Faktor	Unterschiedliche Ausprägung von Schweregrad und Unterschiede in der Mortalität	–
Direktes vs. indirektes ARDS	Unterschiede in der Mortalität und Pathophysiologie	Response auf PEEP wahrscheinlicher bei indirektem ARDS
„Early vs. late onset“	< oder > 48 h nach Aufnahme; Unterschiede in der Pathophysiologie	–
Bildgebung	Diffus vs. fokal Unterschiede in der Atemmechanik und Mortalität	Response auf PEEP wahrscheinlicher bei diffusen Infiltraten
Genetische Faktoren	Genetische Variabilität Unterschiede in Risiko, Prognose, Therapieansprechen	–
Biomarker	z. B. Inflammationsparameter; Unterschiede in Risiko, Prognose, Therapieansprechen	–
Inflammation	Hyperinflammatorisch (höhere inflammatorische Biomarker, höhere Mortalität) vs. hypoinflammatorisch	Höherer PEEP und konservatives Volumenmanagement bei Hyperinflammation
Atemmechanik	Rekrutierbare vs. nichtrekrutierbare Lungenareale; Compliance bzw. Elastizität des respiratorischen Systems; Pleuradruck bzw. transpulmonale Druckverhältnisse	Höherer PEEP bei Rekrutierbarkeit und hohem Pleuradruck

*F*_*i*_*O*_*2*_ inspiratorische Sauerstofffraktion, *p*_*a*_*O*_*2 *_Sauerstoffpartialdruck, *PEEP* positiver endexspiratorischer Druck, *vv-ECMO* venovenöse extrakorporalen Membranoxygenierung

### Schweregrad der Gasaustauschstörung

Die Therapiesteuerung anhand des Schwergrads der Oxygenierungsstörung (mildes, moderates und schweres ARDS) ist seit Jahren etabliert. So wird z. B. Bauchlagerung beim moderatem/schwerem ARDS mit einer p_a_O_2_-F_i_O2-Ratio von < 150 empfohlen. Neu ist die Etablierung des extrakorporalen Lungenersatzes (vv-ECMO) als Standardtherapie bei schwerstem ARDS mit einer p_a_O_2_-F_i_O_2_-Ratio von ≤ 80 (bzw. einer schweren respiratorischen Acidose) auf Basis der Erfahrungen aus der COVID-Pandemie sowie positiver Evidenz [9]. Eine weitere technische und medizinische Optimierung dieser Therapie (unter Beachtung der Kontraindikationen) wird der besseren Steuerung des ECMO-Einsatzes dienen. Die ECMO verbessert nicht nur die Oxygenierung sondern trägt auch zu einer Entlastung des respiratorischen Systems durch Rücknahme der Beatmungsinvasivität bei und führt so zu einer Reduktion der inflammatorischen Antwort des Organismus und der sekundären Organversagen [10, 11].

### Inflammation

Das ARDS ist ein inflammatorisches Syndrom; dementsprechend bietet sich die Modulation der entzündlichen Kaskaden an, um die Progression der Erkrankung zu verhindern. Seit Jahren werden Steroide eingesetzt, deren Wirksamkeit bei CARDS belegt, bei nicht-COVID-19-assoziiertem ARDS (Non-CARDS) allerdings nach wie vor umstritten ist. Bei direktem (pulmonalem) ARDS ausgelöst durch Pneumonie („community acquired pneumonia“) führt eine moderat dosierte Steroidtherapie (analog einer Steroidtherapie bei septischem Schock) zu einem Überlebensvorteil [12]. Wie bei der Sepsis scheint hier die Kombination aus Hydrokortison und Fludrokortison am besten abzuschneiden [13]. Immunmodulationen durch Inhibitoren der Januskinase (JAK) oder Interleukin(IL)-Hemmung (Tozilizumab) wurden beim CARDS mit umstrittenem Erfolg eingesetzt, beim Non-CARDS fehlen bislang Daten dazu. Analog zur Behandlung von Patient:innen im septischen Schock wurde die extrakorporale Zytokinelimination bei CARDS-Patient:innen unter ECMO evaluiert, eine kleine randomisierte Studie zeigte allerdings eine signifikant höhere Mortalität in der Interventionsgruppe [14]. Dass das Ausmaß der inflammatorischen Antwort auf einen auslösenden Trigger Relevanz für den Verlauf der Erkrankung hat, zeigen zumindest Daten zum unterschiedlichen Ansprechen auf therapeutische Interventionen (s. im Folgenden).

### Atemmechanik

Seit mehr als 25 Jahren gilt die sog. protektive Beatmung als Goldstandard in der supportiven ARDS-Therapie. Die ARMA-Studie des ARDS Network zeigte damals einen signifikanten Überlebensvorteil zugunsten einer Beatmung mit 6 vs. 13 ml/kg ideales Körpergewicht, indem VILI verhindert werden konnte. Die VILI entsteht aus „stress and strain“ (mechanische Spannung und Dehnung) an den Alveolen, d. h. aus der durch Überdehnung (Volutrauma), zyklische Recruitments (Atelekttrauma) sowie der durch die auf die Lunge einwirkenden Kräfte entstehenden zusätzlichen entzündlichen Komponente (Biotrauma). Protektive Beatmung soll demnach dazu beitragen, eine Überdehnung durch niedrige Tidalvolumina (und damit möglichst niedrige Atemwegsdrücke), ein zyklischen Recruitment durch Rekrutierung und Offenhalten der Alveolen durch adäquaten PEEP sowie ein Biotrauma durch daraus resultierend möglichst wenig „stress and strain“ zu vermeiden. Das Problem liegt darin, in Anbetracht der inter- und intraindividuellen Heterogenität (unterschiedliche Alveolarbezirke innerhalb einer schwerkranken Lunge) Tools zu identifizieren, die eine solche Beatmung ermöglichen. Luciano Gattinoni hat bereits vor vielen Jahren nachgewiesen, dass sich die Rekrutierbarkeit von atelektatischen Alveolarbezirken je nach zugrunde liegender Pathophysiologie interindividuell unterschiedlich verhält, was die atemmechanischen Eigenschaften unter Beatmung natürlich stark beeinflusst. Fehlende Rekrutierbarkeit bedeutet z. B., dass hohe Atemwegsdrücke inklusive eines hohen PEEP zu wenig Zugewinn an Alveolarfläche führen und dass allerdings das Risiko einer Überdehnung steigt [15].

**Abb. 1:**
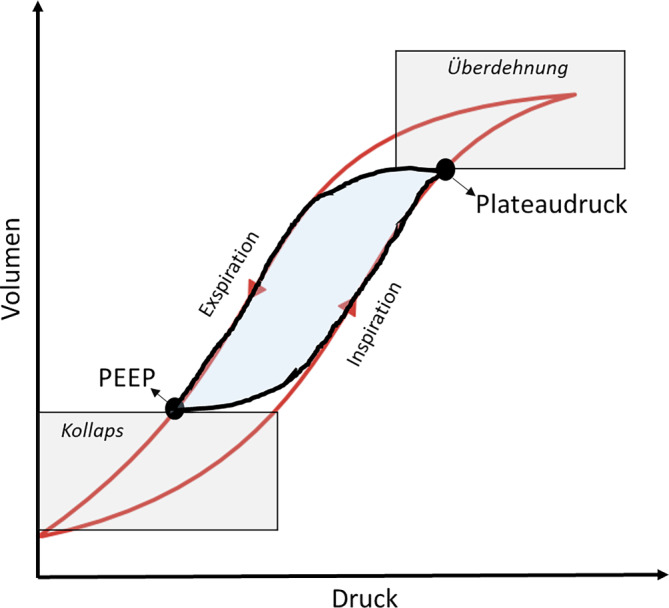
Druck-Volumen-Beziehung bei Positivdruckbeatmung. Der *schwarz* umrandete *blau* gefüllte Bereich stellt die Grenzen einer optimierten „protektiven“ Beatmung dar. *PEEP* positiver endexspiratorischer Druck. (Aus [5])

Die Druck-Volumen-Beziehung zeigt, dass inspiratorisch höhere Atemwegsdrücke zur Dehnung der Lunge nötiger sind als exspiratorisch, um die Alveolen offen, d. h. luftgefüllt, zu halten **(**Abb. 1**)**. Daraus ergibt sich in der Inspiration die Notwendigkeit eines Druckanstiegs, der jedoch nicht zur endtidalen Überdehnung führen soll, und exspiratorisch eines adäquaten PEEP, um einen Alveolarkollaps zu vermeiden.

Wir befinden uns solchermaßen also im steilen Teil der Druck-Volumen-Beziehung und damit im Bereich der höchsten Compliance (= höchste Volumenänderung bei geringster Druckänderung). Daraus ergibt sich der für ein vorgegebenes Tidalvolumen geringste „driving pressure“ (Druckamplitude zwischen Endexpiration und Plateaudruck). Der „driving pressure“ korreliert deshalb besser mit dem Anspruch einer protektiven Beatmung als der Plateaudruck [16]. Trotz des Fehlens konkreter Empfehlungen darf aus diesen pathophysiologischen Erwägungen heraus nicht der Eindruck entstehen, dass eine Titration des PEEP oder dessen Höhe keine Rolle spielt. Es ist zwar richtig, dass die Evidenz hinsichtlich des Endpunkts Mortalität schwach ist, die verfügbaren Studien vergleichen allerdings z. B. nichtindividuelle PEEP-Strategien anhand F_i_O_2_-PEEP-Tabellen. Hier wurde kein Überlebensvorteil zugunsten höherer PEEP-Niveaus als in den ursprünglichen ARDS-Network-Studien gefunden [17]. Eine fixe Vorgabe des PEEP-Niveaus anhand des Sauerstoffbedarfs birgt allerdings die Gefahr einerseits einer insuffizienten Rekrutierung (z. B. bei hohem Pleuradruck) und andererseits einer Überdehnung (z. B. bei fehlender Rekrutierbarkeit), was den Nettoeffekt auf die Mortalität wesentlich beeinflussen könnte. In einer Studie, die PEEP-Niveaus auf Basis der Compliance des respiratorischen Systems titriert hat („EXPRESS-Protokoll“), wurde im Vergleich zu niedrigen PEEP-Niveaus zumindest ein Vorteil hinsichtlich Gasaustausch, Beatmungsdauer und Organversagen nachgewiesen [18]. Eine Metaanalyse zeigte eine Mortalitätsverbesserung zugunsten höhere PEEP-Niveaus bei moderatem und schwerem ARDS, aber den umgekehrten Effekt bei mildem ARDS [19]; eine zweite rezentere Analyse ebenfalls einen möglichen Vorteil für höhere PEEP-Niveaus [20].

Eine individuelle PEEP-Titration ist aus pathophysiologischen atemmechanisch orientierten Überlegungen sinnvoll. Die Routinetitration anhand der Compliance nach prolongierten Recruitmentmanövern birgt allerdings Gefahren und sollte vermieden werden [21]. Das Konzept des transpulmonalen (auch: transalveolären) Drucks (P_L_) trägt dazu bei, sich diesen „protektiven“ Atemwegsdrücken anzunähern: Eine Alveole kann nur offen, d. h. luftgefüllt, sein, wenn der Alveolardruck höher ist als der von außen auf diese Alveole wirkende Druck (sog. Pleuradruck). Die Messung des transpulmonalen Drucks mittels Ösophagusdrucksonde lässt eine Individualisierung der Beatmung hinsichtlich PEEP-Titration und Vermeidung von Überdehnung zu, allerdings ist die Studienlage zu den Effekten auf Outcomeparameter widersprüchlich [22, 23]. Ähnliches gilt für die elektrische Impedanztomographie (EIT), die Ventilation, Rekrutierbarkeit und Überdehnung in Echtzeit von Atemhub zu Atemhub erfasst. Eine Individualisierung der Beatmung anhand dieser Parameter führt zu positiven atemmechanischen Effekten und damit potenziell zur Reduktion von VILI [24].

Da letztlich VILI ein Resultat der auf die Lunge wirkenden Energie darstellt, könnte die Beatmungsleistung („mechanical power of ventilation“) einen geeigneten Zielparameter darstellen, um die schädliche Einwirkung von Positivdruckbeatmung auf die Lunge zu minimieren. In einer großen retrospektiven Analyse von ARDS-Patient:innen wurde ein Zusammenhang zwischen steigender Beatmungsleistung und Mortalität ermittelt [25]. Die Beatmungsleistung wird in J/min angegeben und kann nach folgenden Formeln annäherungsweise berechnet werden (wobei P_peak_ für den inspiratorischen Spitzendruck, V_T_ für Tidalvolumen und AF für Atemfrequenz steht):

- Volumenorientierte Beatmung: 0,1 × (P_peak_ − 0,5 × ∆P) × VT × AF
- Druckorientierte Beatmung: 0,1 × P_peak_ × VT × AF

Es besteht somit reichlich Potenzial, über das 25 Jahre alte Konzept der „Lungenprotektion“ durch niedrige Tidalvolumina hinaus die Beatmungstherapie zu optimieren und zu individualisieren.

## Heterogenität des ARDS als Basis des individualisierten Therapiemanagements

In der Intensivmedizin werden große Hoffnungen in neue ARDS-Strategien, die zunächst in kleineren Studien noch positive Effekte zeigten [26, 27], oftmals durch darauffolgende adäquat gepowerte Studien durch einen fehlenden klinischen Benefit in einem heterogenen Patientengut zerstört. Als Beispiele hierfür gelten der ROSE-Trial ([28]; keine Verbesserung der 90-Tage-Mortalität durch kontinuierliche neuromuskuläre Blockade) oder der HARP-2-Trial ([29]; keine Verbesserung von klinischen Endpunkten durch Simvastatin). Die oft unbeantwortete Frage ist, ob in diesen Kohorten nicht doch Subgruppen existieren, die von ARDS-spezifischen Interventionen profitieren könnten.

### Unterscheidung von ARDS-Subtypen durch „latent class analysis“

Einen sehr interessanten Ansatz zur Unterscheidung von ARDS-Subtypen bietet die statistische Methode der *„latent class analysis“* zur Identifikation von einzelnen Gruppen innerhalb eines heterogenen Kollektivs, die ähnliche Eigenschaften bezüglich beobachtbarer Variablen aufweisen. Diese Variablen können demografische (z. B. Alter, Geschlecht), klinische (z. B. Sequential-organ-failure -assessment[SOFA]-Score-Domänen), beatmungsassoziierte (z. B. „driving pressure“ oder Tidalvolumina), biologische (z. B. Zytokinspiegel, inflammatorische Biomarker) oder Outcomeparameter (z. B. Mortalität, Beatmungsdauer) umfassen [30].

Mithilfe dieser statistischen Methode wurden in der Patientenkohorte des ARMA-Trials und des ALVEOLI-Trials mit einem hyper- und einem hypoinflammatorischen Typ 2 ARDS-Subgruppen unterschieden und beschrieben. Der hyperinflammatorische Subtyp zeichnete sich durch höhere Plasmaspiegel von inflammatorischen Biomarkern, höheren Vasopressorensupport, niedrigere Serumbikarbonatkonzentration und höhere Prävalenz von Sepsis aus. Der hyperinflammatorische Typ zeigte hierbei in beiden Kohorten eine signifikant höhere Mortalität und mehr Tage mit Organversagen [30].

### Unterschiedliches Ansprechen von ARDS-Subtypen auf Interventionen

In der ALVEOLI-Kohorte wurde mit der Unterteilung in einen hyper- und hypoinflammatorischen Subtyp nachgewiesen, dass eine High-PEEP-Strategie bei Patient:innen mit einem hyperinflammatorischen Typ Mortalität und Tage mit Organversagen reduziert, während in der hypoinflammatorischen Subgruppe ein gegenteiliger Effekt zu sehen war [30]. Dieselbe Methode wurde auch für eine sekundäre Analyse des HARP-2 Trials (Simvastatin in ARDS) angewandt [31]. Auch hier zeigte sich, dass die Simvastatingabe einen statistisch signifikanten Benefit bei Patient:innen mit hyperinflammatorischem ARDS, jedoch nicht beim hypoinflammatorischen Typ erzielt.

### ARDS-Trials der Gegenwart und Zukunft

*„Population enrichment methods“ *(im deutschen am ehesten als Strategien zur Selektion spezifischer Patientengruppen zu übersetzen) ist das Stichwort der Gegenwart und Zukunft von ARDS-Trials. *„Enrichment“* bezeichnet hierbei die Selektion einer Patientenkohorte, die am ehesten von einer Intervention profitiert [32], *„prognostic enrichment“ *die Selektion von Patient:innen, die das höchste Risiko für das Erreichen eines krankheitsspezifischen klinischen Endpunkt haben – z. B. die höchste ARDS-assoziierte Mortalität. *„Prognostic enrichment“ *reduziert zudem die notwendige Stichprobengröße, um eine relative Risikoreduktion für das Erreichen eines klinischen Endpunkts zu erzielen, allerdings ohne die Heterogenität einer Patientenkohorte zu addressieren [33].

Letzteres wird durch *„predictive enrichment“ *erreicht. Diese Strategie zielt darauf ab, Patient:innen anhand von klinischer Erscheinung oder Biomarkern – wie bei den hyper- und hypoinflammatorischen ARDS-Subtypen – zu identifizieren, die am ehesten von Interventionen profitieren [33].

### Neuen Studiendesigns – adaptive „Platform-Trials“

**Abb. 2:**
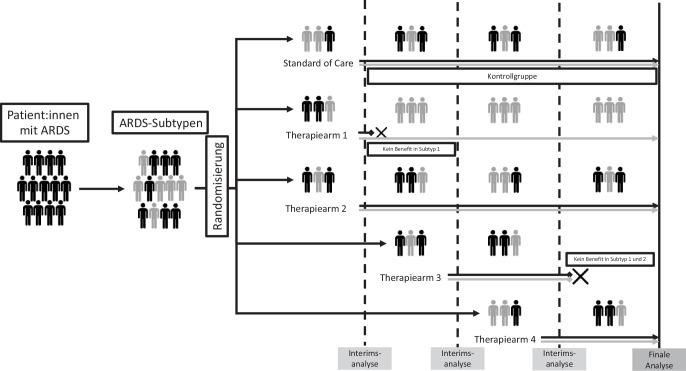
Ablauf von Platform-Trials. *ARDS* Atemnotsyndrom

Neben *Enrichment*-Strategien werden innovative Trialdesigns die Zukunft der intensivmedizinischen Studienlandschaft prägen. Die sog. *Platform-Trials* basieren hierbei auf folgenden Prinzipien (Abb. 2; [34]):

- *Flexibilität:* es können mehrere Interventionen (Medikamente oder Therapieansätze) gleichzeitig getestet werden.
- *Adaptives Design:* Neue Interventionen können dynamisch hinzugefügt werden, ohne dass hierfür die Studie pausiert oder unterbrochen werden muss. Ebenso können Therapiearme nach Maßgabe von Zwischenergebnissen gestoppt werden.
- *Gemeinsame Kontrollgruppe:* Trotz mehrerer Therapiearme ist nur eine Kontrollgruppe notwendig, wodurch die Zahl der Patient:innen, die ausschließlich Placebo bzw. die Standardbehandlung erhalten, reduziert wird.

Um Platform-Trials somit effizient zu gestalten, ist eine optimale Organisationsstruktur unabdingbar. Ein multizentrisches Design und eine langfristige Laufzeit sind für die Durchführung essenziell. Die Planung der Trials erfordert spezialisierte statistische Modelle und der kontinuierliche Einschluss und die Anpassung der Interventionen erfordert eine enge Zusammenarbeit mit Zulassungsbehörden. Somit erfordern Platform-Trials umfangreiche Ressourcen und eine entsprechend nachhaltige, langfristige Finanzierung [35].

Während große Platform-Trials für verschiedene medizinische Krankheitsbilder bereits seit Jahren rekrutieren [36, 37], hat in Bezug auf die intensivmedizinische und insbesondere die ARDS-Forschung vor allem die COVID-19-Pandemie diese Form der Studiendurchführung auf ein neues Level gebracht. Im Zuge der COVID-19-Pandemie hat REMAP-CAP hier zahlreiche Interventionen getestet – wobei das Studiendesign schon vor der Pandemie für Patient:innen mit *„community acquired pneumonia“* geplant wurde [38]. Das RECOVERY-Trial, das für COVID-19-Patient:innen geplant wurde und nunmehr auf Influenza und nichtviral bedingte *„community acquired pneumonia“* ausgeweitet wurde, zeigte außerdem, dass Platform-Trials im Zuge einer Notsituation wie einer Pandemie eine Möglichkeit zur schnellen und effizienten Durchführung von randomisierten kontrollierten Studien bieten [39]. Das PANTHER-Trial soll in diesem Kontext demnächst ein Platform-Trial zur Evaluation von Interventionen bei Patient:innen mit akutem hypoxischem Lungenversagen darstellen. Hierbei werden auch Predictive-enrichment-Strategien verwendet (hyper- und hypoinflammatorischer ARDS-Subtyp). Die ersten beiden Interventionsarme werden Simvastatin und Baricitinib darstellen [40].

## Stellenwert der künstlichen Intelligenz beim ARDS-Management

Vor etwa 15 Jahren wurden die ersten Publikationen zur künstlichen Intelligenz (KI) bei ARDS veröffentlicht. Seither wurden verschiedenste KI-basierte Verfahren geprüft. Am häufigsten kommt hierbei das Machine Learning zum Einsatz, das Computern ermöglicht, aus Daten zu lernen und ohne explizite Programmierung Entscheidungen oder Vorhersagen zu treffen. Statt feste Regeln vorzugeben, entwickelt der Computer Modelle, die Muster und Zusammenhänge in Daten erkennen und nutzen können. Beim Machine Learning können verschiedene Arten des Lernens angewandt werden. In der Medizin handelt es sich hierbei meistens um die Methode des *Supervised Learnings*, bei der das Modell aus einem Datensatz mit bekannten Ergebnissen lernt [41].

Neuronale Netzwerke können der Erkennung von komplexen Mustern in großen, multidimensionalen Datensätzen, z. B. anhand von Lungencomputertomographiebildern, Beatmungsparametern oder genetischen Informationen, dienen. Sie sind besonders nützlich, um subtile Zusammenhänge zu identifizieren, die mit traditionellen Methoden schwer zu erfassen sind, und ermöglichen so präzisere Diagnosen und Therapieentscheidungen.

Aufgrund der hohen Relevanz therapierelevanter Entscheidungen, die in der medizinischen Versorgung tagtäglich von Intensivmediziner:innen getroffen werden müssen, ist es unwahrscheinlich, dass diese in naher Zukunft allein KI-basiert getroffen werden [42]. Allerdings verfügen wir mit der heutzutage verwendbaren KI über ein Hilfsmittel, das Mediziner:innen hervorragend unterstützen und unsere Arbeit effizienter machen kann (Tab. 4; [41]).

**Tab. 4:** Anwendungsgebiete von künstlicher Intelligenz mit Vor- und Nachteilen beim Atemnotsyndrom (*ARDS*)

Anwendungsgebiet	Vorteile	Nachteile
*Früherkennung und Diagnose*	Schnelle und präzise Analyse von Röntgenbildern oder Computertomographien	Abhängigkeit von hochwertigen Daten
	Vorhersage der ARDS-Entwicklung in hospitalisierten Patient:innen	Risiko von Fehldiagnosen bei unausgewogenen Datensätzen
*Patientenstratifizierung*	Identifikation von ARDS-Phänotypen (z. B. hyperinflammatorisch)	Erfordernis von umfangreichen Biomarker- und klinischen Daten
	Risikoabschätzung für Mortalität	Erhöhte Komplexität in der klinischen Anwendung
*Personalisierte Therapie*	Optimierung von Behandlungsstrategien	Schwierige Validierung von Modellen in der klinischen Praxis
	Verbesserung der Beatmungseinstellungen	Gefahr von Überanpassung („overfitting“)
*Überwachung und Prognose*	Echtzeitüberwachung von Vitalparametern	Mögliche Fehlalarme durch Algorithmusungenauigkeiten
	Vorhersage von Krankheitsverlauf und Komplikationen	Erfordernis einer stabilen technischen Infrastruktur
*Forschung und klinische Studien*	Effiziente Patientenrekrutierung	Hohe organisatorische und technische Anforderungen
	Anpassung von Studien in Echtzeit (z. B. in Platform-Trials)	Komplexe regulatorische Hürden
*Big-Data-Analyse*	Integration und Analyse großer, heterogener Datensätze	Hoher Rechenaufwand
	Entdeckung neuer Biomarker	Schwierigkeit, Daten zu standardisieren und zu harmonisieren

### Radiologische KI-basierte ARDS-Früherkennung

Die KI-Anwendung hat großes Potenzial in der (radiologischen) Früherkennung des ARDS. In der LUNG-SAFE-Studie wurde nachgewiesen, dass nur rund die Hälfte der Patient:innen mit mildem ARDS als solche identifiziert werden. Bei Patient:innen mit schwerem ARDS wurden immerhin noch etwa 20 % nicht als solche identifiziert. In einer rezenten Studie wurde gezeigt, dass naturgemäß ARDS weder von Radiolog:innen noch mithilfe von KI in Röntgenbildern sicher richtig klassifiziert werden kann (80,8 % bzw. 84,7 % Genauigkeit). In dieser Studie wurde allerdings demonstriert, dass bei einer unterstützenden Methode, in der zunächst alle Rötgenbilder KI-basiert analysiert und lediglich die, bei denen hierbei Unsicherheit besteht (was etwa jedes 5. Röntgenbild betrifft), ärztlich beurteilt werden, die Genauigkeit der Identifikation auf 87 % steigt – dabei allerdings eben nur jedes 5. Bild manuell begutachtet werden muss [43]. Mit einem derartigen KI-gestützten Ansatz könnte also sowohl die Genauigkeit der Beurteilung gesteigert als auch die Arbeitslast deutlich reduziert werden.

### Weitere KI-Anwendungen bei ARDS

Zusätzlich könnten in Zukunft auch KI-gestützte Modelle zur Vorhersage der individuellen ARDS-Mortalität genutzt werden, was wiederum beim zuvor erwähnten *„prognostic enrichment“* eingesetzt werden kann [44]. *„Decision-support-Systeme“*, die Intensivmediziner:innen in der Wahl der Beatmungseinstellungen von ARDS-Patient:innen unterstützen könnten, scheinen ein naheliegendes Tool zur Optimierung der Behandlung zu sein. In einer kürzlich veröffentlichten randomisierten kontrollierten Studie, in der ein Decision-support-System als Intervention getestet wurde, wurde allerdings kein Vorteil im primären Endpunkt – die Reduktion des „driving pressure“, erzielt werden [45].

Zusammenfassend wird in Zukunft unsere intensivmedizinische Arbeit definitiv nicht durch KI ersetzt werden. Dennoch bietet die Nutzung von KI vielversprechende Möglichkeiten, um Entscheidungsfindungen sicherer und effizienter zu gestalten und durchaus auch Arbeitslast zu reduzieren, was in Anbetracht der dünnen Personaldecke in vielen Kliniken einen großen Mehrwert darstellen könnte.

## Fazit für die Praxis

- Die Behandlung des Atemnotsyndroms (ARDS) hat sich in den letzten Jahren durch technologische Innovationen und ein besseres Verständnis der Pathophysiologie weiterentwickelt.
- Ein individueller, patientenfokussierter Ansatz, unterstützt durch neue Leitlinien, bildgebende Verfahren und künstliche Intelligenz, wird zur weiteren Verbesserung der Prognose entscheidend sein.
- Innovative Therapieansätze bieten vielversprechende Möglichkeiten, die Morbidität und Mortalität dieser komplexen Erkrankung weiter zu reduzieren.

## References

[1] Maslove DM, Tang B, Shankar-Hari M et al (2022) Redefining critical illness. Nat Med 28(6):1141–1148 35715504 10.1038/s41591-022-01843-x

[2] Grasselli G, Calfee CS, Camporota L et al (2023) ESICM guidelines on acute respiratory distress syndrome: definition, phenotyping and respiratory support strategies. Intensive Care Med 49(7):727–759 37326646 10.1007/s00134-023-07050-7 PMC10354163

[3] Qadir N, Sahetya S, Munshi L et al (2024) An update on management of adult patients with acute respiratory distress syndrome: an official American thoracic society clinical practice guideline. Am J Respir Crit Care Med 209(1):24–36 38032683 10.1164/rccm.202311-2011ST PMC10870893

[4] Matthay MA, Arabi Y, Arroliga AC et al (2024) A new global definition of acute respiratory distress syndrome. Am J Respir Crit Care Med 209(1):37–47 37487152 10.1164/rccm.202303-0558WS PMC10870872

[5] Staudinger T (2025) Acute respiratory distress syndrome : pathophysiology, definition and treatment strategies. Med Klin Intensivmed Notfmed 120(1):81–93 39777483 10.1007/s00063-024-01218-9

[6] Neetz B, Flohr T, Herth FJF et al (2021) Patient self-inflicted lung injury (P-SILI) : from pathophysiology to clinical evaluation with differentiated management. Med Klin Intensivmed Notfmed 116(7):614–623 33961061 10.1007/s00063-021-00823-2 PMC8103432

[7] Bellani G, Laffey JG, Pham T et al (2017) Noninvasive ventilation of patients with acute respiratory distress syndrome insights from the LUNG SAFE study. Am J Respir Crit Care Med 195(1):67–77 27753501 10.1164/rccm.201606-1306OC

[8] Grieco DL, Menga LS, Eleuteri D et al (2019) Patient self-inflicted lung injury: implications for acute hypoxemic respiratory failure and ARDS patients on non-invasive support. Minerva Anestesiol 85(9):1014–1023 30871304 10.23736/S0375-9393.19.13418-9

[9] Combes A, Peek GJ, Hajage D et al (2020) ECMO for severe ARDS: systematic review and individual patient data meta-analysis. Intensive Care Med 46(11):2048–2057 33021684 10.1007/s00134-020-06248-3 PMC7537368

[10] Combes A, Hajage D, Capellier G et al (2018) Extracorporeal membrane oxygenation for severe acute respiratory distress syndrome. N Engl J Med 378(21):1965–1975 29791822 10.1056/NEJMoa1800385

[11] Burrell AJC, Lubnow M, Enger TB et al (2017) The impact of venovenous extracorporeal membrane oxygenation on cytokine levels in patients with severe acute respiratory distress syndrome: a prospective, observational study. Crit Care Resusc 19(1):37–44 29084500

[12] Dequin PF, Meziani F, Le Gouge A (2023) Hydrocortisone in severe community-acquired pneumonia. Reply. N Engl J Med 389(7):671–672 37585640 10.1056/NEJMc2307400

[13] Heming N, Renault A, Kuperminc E et al (2024) Hydrocortisone plus fludrocortisone for community acquired pneumonia-related septic shock: a subgroup analysis of the APROCCHSS phase 3 randomised trial. Lancet Respir Med 12(5):366–374 38310918 10.1016/S2213-2600(23)00430-7

[14] Supady A, Weber E, Rieder M et al (2021) Cytokine adsorption in patients with severe COVID-19 pneumonia requiring extracorporeal membrane oxygenation (CYCOV): a single centre, open-label, randomised, controlled trial. Lancet Respir Med 9(7):755–762 34000236 10.1016/S2213-2600(21)00177-6 PMC8121541

[15] Gattinoni L, Caironi P, Cressoni M et al (2006) Lung recruitment in patients with the acute respiratory distress syndrome. N Engl J Med 354(17):1775–1786 16641394 10.1056/NEJMoa052052

[16] Amato MB, Meade MO, Slutsky AS et al (2015) Driving pressure and survival in the acute respiratory distress syndrome. N Engl J Med 372(8):747–755 25693014 10.1056/NEJMsa1410639

[17] Brower RG, Lanken PN, MacIntyre N et al (2004) Higher versus lower positive end-expiratory pressures in patients with the acute respiratory distress syndrome. N Engl J Med 351(4):327–336 15269312 10.1056/NEJMoa032193

[18] Mercat A, Richard JC, Vielle B et al (2008) Positive end-expiratory pressure setting in adults with acute lung injury and acute respiratory distress syndrome: a randomized controlled trial. JAMA 299(6):646–655 18270353 10.1001/jama.299.6.646

[19] Briel M, Meade M, Mercat A et al (2010) Higher vs lower positive end-expiratory pressure in patients with acute lung injury and acute respiratory distress syndrome: systematic review and meta-analysis. JAMA 303(9):865–873 20197533 10.1001/jama.2010.218

[20] Dianti J, Tisminetzky M, Ferreyro BL et al (2022) Association of positive end-expiratory pressure and lung recruitment selection strategies with mortality in acute respiratory distress syndrome: a systematic review and network meta-analysis. Am J Respir Crit Care Med 205(11):1300–1310 35180042 10.1164/rccm.202108-1972OC PMC12042658

[21] Writing Group for the Alveolar Recruitment for Acute Respiratory Distress Syndrome Trial I, Cavalcanti AB, Suzumura EA et al (2017) Effect of lung recruitment and titrated positive end-expiratory pressure (PEEP) vs low PEEP on mortality in patients with acute respiratory distress syndrome: a randomized clinical trial. JAMA 318(14):1335–1345 28973363 10.1001/jama.2017.14171 PMC5710484

[22] Talmor D, Sarge T, Malhotra A et al (2008) Mechanical ventilation guided by esophageal pressure in acute lung injury. N Engl J Med 359(20):2095–2104 19001507 10.1056/NEJMoa0708638 PMC3969885

[23] Beitler JR, Sarge T, Banner-Goodspeed VM et al (2019) Effect of titrating positive end-expiratory pressure (PEEP) with an esophageal pressure-guided strategy vs an empirical high PEEP-Fio2 strategy on death and days free from mechanical ventilation among patients with acute respiratory distress syndrome: a randomized clinical trial. JAMA 321(9):846–857 30776290 10.1001/jama.2019.0555 PMC6439595

[24] Songsangvorn N, Xu Y, Lu C et al (2024) Electrical impedance tomography-guided positive end-expiratory pressure titration in ARDS: a systematic review and meta-analysis. Intensive Care Med. 10.1007/s00134-024-07362-2 38512400 10.1007/s00134-024-07362-2 PMC11078723

[25] Serpa Neto A, Deliberato RO, Johnson AEW et al (2018) Mechanical power of ventilation is associated with mortality in critically ill patients: an analysis of patients in two observational cohorts. Intensive Care Med 44(11):1914–1922 30291378 10.1007/s00134-018-5375-6

[26] Papazian L, Forel JM, Gacouin A et al (2010) Neuromuscular blockers in early acute respiratory distress syndrome. N Engl J Med 363(12):1107–1116 20843245 10.1056/NEJMoa1005372

[27] Craig TR, Duffy MJ, Shyamsundar M et al (2011) A randomized clinical trial of hydroxymethylglutaryl-coenzyme a reductase inhibition for acute lung injury (the HARP study). Am J Respir Crit Care Med 183(5):620–626 20870757 10.1164/rccm.201003-0423OC

[28] National Heart L, Blood Institute PCTN, Moss M et al (2019) Early neuromuscular blockade in the acute respiratory distress syndrome. N Engl J Med 380(21):1997–2008 31112383 10.1056/NEJMoa1901686 PMC6741345

[29] McAuley DF, Laffey JG, O’Kane CM et al (2014) Simvastatin in the acute respiratory distress syndrome. N Engl J Med 371(18):1695–1703 25268516 10.1056/NEJMoa1403285

[30] Calfee CS, Delucchi K, Parsons PE et al (2014) Subphenotypes in acute respiratory distress syndrome: latent class analysis of data from two randomised controlled trials. Lancet Respir Med 2(8):611–620 24853585 10.1016/S2213-2600(14)70097-9 PMC4154544

[31] Calfee CS, Delucchi KL, Sinha P et al (2018) Acute respiratory distress syndrome subphenotypes and differential response to simvastatin: secondary analysis of a randomised controlled trial. Lancet Respir Med 6(9):691–698 30078618 10.1016/S2213-2600(18)30177-2 PMC6201750

[32] Stanski NL, Wong HR (2020) Prognostic and predictive enrichment in sepsis. Nat Rev Nephrol 16(1):20–31 31511662 10.1038/s41581-019-0199-3 PMC7097452

[33] Beitler JR, Thompson BT, Baron RM et al (2022) Advancing precision medicine for acute respiratory distress syndrome. Lancet Respir Med 10(1):107–120 34310901 10.1016/S2213-2600(21)00157-0 PMC8302189

[34] Berry SM, Connor JT, Lewis RJ (2015) The platform trial: an efficient strategy for evaluating multiple treatments. JAMA 313(16):1619–1620 25799162 10.1001/jama.2015.2316

[35] Woodcock J, LaVange LM (2017) Master protocols to study multiple therapies, multiple diseases, or both. N Engl J Med 377(1):62–70 28679092 10.1056/NEJMra1510062

[36] Barker AD, Sigman CC, Kelloff GJ et al (2009) I‑SPY 2: an adaptive breast cancer trial design in the setting of neoadjuvant chemotherapy. Clin Pharmacol Ther 86(1):97–100 19440188 10.1038/clpt.2009.68

[37] Israel E, Denlinger LC, Bacharier LB et al (2021) PrecISE: precision medicine in severe asthma: an adaptive platform trial with biomarker ascertainment. J Allergy Clin Immunol 147(5):1594–1601 33667479 10.1016/j.jaci.2021.01.037 PMC8113113

[38] REMAP-CAP trial. https://www.remapcap.org/

[39] RECOVERY trial. https://www.recoverytrial.net/

[40] PANTHER trial. https://panthertrial.org/about

[41] Tran TK, Tran MC, Joseph A et al (2024) A systematic review of machine learning models for management, prediction and classification of ARDS. Respir Res 25(1):232 38834976 10.1186/s12931-024-02834-x PMC11151485

[42] Topol EJ (2019) High-performance medicine: the convergence of human and artificial intelligence. Nat Med 25(1):44–56 30617339 10.1038/s41591-018-0300-7

[43] Farzaneh N, Ansari S, Lee E et al (2023) Collaborative strategies for deploying artificial intelligence to complement physician diagnoses of acute respiratory distress syndrome. NPJ Digit Med 6(1):62 37031252 10.1038/s41746-023-00797-9 PMC10082784

[44] Rashid M, Ramakrishnan M, Chandran VP et al (2022) Artificial intelligence in acute respiratory distress syndrome: a systematic review. Artif Intell Med 131:102361 36100348 10.1016/j.artmed.2022.102361

[45] Patel BV, Mumby S, Johnson N et al (2024) A randomized control trial evaluating the advice of a physiological-model/digital twin-based decision support system on mechanical ventilation in patients with acute respiratory distress syndrome. Front Med 11:1473629 10.3389/fmed.2024.1473629 PMC11559429 39540041

